# Comparative analysis of affinity-based 5-hydroxymethylation enrichment techniques

**DOI:** 10.1093/nar/gkt1080

**Published:** 2013-11-07

**Authors:** John P. Thomson, Jennifer M. Hunter, Colm E. Nestor, Donncha S. Dunican, Rémi Terranova, Jonathan G. Moggs, Richard R. Meehan

**Affiliations:** ^1^Chromosomes and Gene Expression, MRC Human Genetics Unit at the Institute of Genetics and Molecular Medicine at the University of Edinburgh, Crewe Road, Edinburgh EH4 2XU, UK, ^2^Member of MARCAR Consortium, ^3^The Centre for Individualized Medication, Linköping University Hospital, Linköping University, Linköping SE-58185, Sweden and ^4^Discovery and Investigative Safety, Preclinical Safety, Novartis Institutes for Biomedical Research, Klybeckstrasse, Basel CH-4002, Switzerland

## Abstract

The epigenetic modification of 5-hydroxymethylcytosine (5hmC) is receiving great attention due to its potential role in DNA methylation reprogramming and as a cell state identifier. Given this interest, it is important to identify reliable and cost-effective methods for the enrichment of 5hmC marked DNA for downstream analysis. We tested three commonly used affinity-based enrichment techniques; (i) antibody, (ii) chemical capture and (iii) protein affinity enrichment and assessed their ability to accurately and reproducibly report 5hmC profiles in mouse tissues containing high (brain) and lower (liver) levels of 5hmC. The protein-affinity technique is a poor reporter of 5hmC profiles, delivering 5hmC patterns that are incompatible with other methods. Both antibody and chemical capture-based techniques generate highly similar genome-wide patterns for 5hmC, which are independently validated by standard quantitative PCR (qPCR) and glucosyl-sensitive restriction enzyme digestion (gRES-qPCR). Both antibody and chemical capture generated profiles reproducibly link to unique chromatin modification profiles associated with 5hmC. However, there appears to be a slight bias of the antibody to bind to regions of DNA rich in simple repeats. Ultimately, the increased specificity observed with chemical capture-based approaches makes this an attractive method for the analysis of locus-specific or genome-wide patterns of 5hmC.

## INTRODUCTION

Direct chemical modification of cytosine bases found in the dinucleotide sequence CpG is a common method of epigenetic regulation in the mammalian genome (1). The most common form of this modification is the addition of a methyl group to the carbon 5 position on the pyrimidine ring by a family of DNA methlytransferase enzymes (Dnmt1, Dnmt3a and Dnmt3b) to form 5-methylcytosine (5mC). Methylation events are thought to be of critical importance in the silencing of the many repetitive elements found in mammalian genomes as well as having proposed roles in the regulation of imprinting, X-inactivation, constraining polycomb repressor complex (PRC2) targeting of H3K27me3 and general promoter activity (2–4). Recently, there has been renewed interest in the field of DNA methylation due to the identification of a novel set of modified cytosine bases, all of which are found over CpG dinucleotides albeit at far lower abundances than the 5mC modification (5). The most prevalent of these modified bases, corresponding to <1% of cytosine bases in mouse and human tissues, is that of 5-hydroxymethylcytosine (5hmC). Although initially discovered more than 60 years ago, this modification has only recently been comprehensively studied in mammalian genomes (for a review see (6)) and has since been proposed to be part of a demethylation pathway due to the finding that it is formed through oxidation of a methyl group into a hydroxymethyl group at cytosine bases by the Tet-eleven-translocation (TET) family of Fe(II) and α-KG-dependent dioxygenases (Tet1, Tet2 and Tet3) (7–10). These same enzymes have also been shown to further convert the 5hmC-modified cytosines to the derivatives 5-formylcytosine (5fC) and/or 5-carboxylcytosine (5caC), which have been proposed to ultimately result in base excision repair (BER) and replacement with a non-modified cytosine base (11–14). Disruption of the TET proteins has been reported to result in globally reduced 5hmC levels, a phenomenon also seen during carcinogenesis (15). In addition, knockdown of TET1 in embryonic stem cells (ESCs) leads to an increase in 5mC over transcriptional start sites (TSSs) alongside loss of 5hmC at specific promoters and within gene bodies of TET1 target genes (16–18). Knockdown of Tet2 in hematopoietic progenitor cells was found to perturb the normal gene expression pathways involved in differentiation resulting in a block of myeloid differentiation (19,20) while activation of Tet2 target genes in pre-B cells was also seen to accompany changes in the promoter specific patterns of the 5hmC modification (21). Work in *Xenopus* suggests that the Tet3 CXXC domain is required for its targeting to the promoters of genes that are critical for eye and neural development (22). Deletion of the CXXC DNA binding domain from xlTet3 abolishes its ability to occupy target gene promoters, thereby preventing developmental demethylation that is normally associated with their activation. Recently, an ancestral CXXC protein, IDAX, which became separated from TET2 following chromosomal rearrangement, has been shown to have a role in both recruiting TET2 to target genes and regulating its protein stability, ultimately impacting on its dioxygenase activity (23).

**Figure 1.**
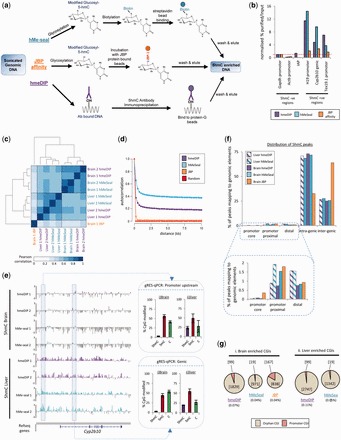
Genome-wide 5hmC patterns in mouse whole brain and liver DNA following enrichment by either antibody, chemical capture or protein affinity-based methods. (**a**) An overview of the three commercially available techniques for 5hmC enrichment. In our study, following enrichment we carried out whole genome amplification and dye labelling for micro-array hybridization. (**b**) qPCR validation of the relative enrichment efficiencies over candidate loci previously identified as being either enriched or depleted in 5hmC in the mouse liver (26). Following normalization to the negative region at the *Gapdh* promoter, all three techniques report similar findings; however, the JBP-1-based affinity technique gives very low enrichment values compared to the hmeDIP and hMeSeal methods. Red dotted line denotes no enrichment over *Gapdh*. (**c**) Pearson correlation analysis and clustering among the microarray datasets. Biological replicates cluster closely while tissues clustered independently confirming the tissue-specific nature of 5hmC patterns. JBP-1 affinity purified 5hmC datasets correlate poorly with the hmeDIP and hMeSeal sets (**d**) Autocorrelation analysis of 5hmC patterns determined by hmeDIP, hMeSeal and JBP-1-binding in a single mouse brain sample. Autocorrelation was determined to a distance of 40 probes (∼10 kb). A ‘random’ sample for comparison was generated by randomization of the hMeSeal data. Filled circles represent relative probe position. (**e**) Example of microarray datasets showing tissue specificity and biological replicate reproducibility between each technique over the liver specific gene *Cyp2b10*. Data are plotted on log2 scales from −3 to +3. Biological replicates are numbered 1 and 2, respectively. Gene structure is shown below by blue bars. Boxed regions are expanded upon on the right to display regions independently validated by gRES-qPCR. Plots represent the percentage of each modification at a single CpG in the sequence CCGG following normalization (purple; 5hmC, red; 5mC, green; C). Error bars display the standard error of the biological replicates. (**f**) Percentage plots of the distributions of 5hmC enriched regions following hmeDIP, hMeSeal and JBP-1 5hmC purification. Peak probes of 5hmC enrichment were defined (see ‘Materials and Methods’ section) and then mapped to one of five unique genomic loci (promoter cores, proximal and distal regions as well as intra- and inter-genic regions; box on right). Red dotted lines highlight changes in the distributions between techniques. Boxed region is expanded upon to reveal technique dependant differences over promoter-core, -proximal and -distal peaks. (**g**) The number and distribution of 5hmC peak probes generated for the three techniques are low over CpG islands (CGI) and largely non-promoter associated. Pie charts representative of the dataset size reveal low numbers of CGI related 5hmC peak probes following hmeDIP or hMeSeal in the brain (i) and the liver (ii). The total number of peaks mapping to CGIs are shown in square brackets while round brackets denote the total per cent of probes on the arrays which overlapped with CGI enriched peaks. Pink = peak probes mapping to promoter CGI regions, brown = peak probes mapping to orphan CGI (non-promoter) regions.

The field of 5hmC research has advanced rapidly over the past 3 years as the number and accuracy of techniques with which to enrich for 5hmC-containing DNA has increased, especially when combined with the affordability of tilling arrays (24–26) and access of next generational sequencing (NGS) technologies (27).Many of the initial genome-wide studies have focused on the enrichment of 5hmC-containing DNA fragments through antibody precipitation-based methods (hmeDIP), which were similar to the already well-established methyl-DNA immunoprecipitation (MeDIP) protocols (17,28,29) (Figure 1a). Although the majority of these studies were carried out on mouse embryonic stem cells (mESCs), which contain relatively low levels of 5hmC, they successfully mapped the genome-wide distribution of the modification, revealing that 5hmC is typically depleted over the TSSs and enriched in the bodies of highly expressed genes. There was also some evidence to suggest that 5hmC was enriched at repetitive elements, particularly those of the Line1 family (28). However, subsequent studies highlighted potential bias of anti-5hmC antibodies for recognizing modification dense regions (30) as well as showing some bias towards CA repeat sequences (31).

Around the same time, several groups also developed affinity-based methods to enrich for 5hmC marked DNA (32–35). The T4 bacteriophage enzyme β-glucosyltransferase (βGT) is central in many of these techniques due to its ability to specifically modify 5-hydroxymethyl modified cytosine bases through glucosylation, yielding the base, glucosyl-5hmC. By including a chemically altered glucose group within this reaction so that subsequently modified glycosyl-5hmC bases can bind biotin, a technique was developed whereby streptavidin purification would result in extremely efficient purification of DNA fragments (termed hydroxymethyl selective chemical labelling or hMeSeal) (32). Unlike hmeDIP, hMeSeal enrichment can be carried out on very small amounts of material (<100 ng starting sample) and does not appear to suffer from the previously reported CpG bias issues described for hmeDIP, as such providing a method suitable for the analysis for 5hmC levels in regions of low CpG density. A similar approach to the hMeSeal technique was also developed whereby βGT glucosylated 5hmC DNA fragments are subjected to an additional reaction with sodium periodate in order to allow the incorporation of two biotin groups (glucosylation, periodate oxidation, biotinylation or GLIB) (30); however, this extra enzymatic step has been known to introduce DNA damage as well as introduce some background effects (5). A third approach based on the affinity of the trypanosome J binding protein 1 (JBP-1) towards glucosylated 5hmC has also been reported (34); however, only one subsequent study has utilized this technology, reporting little to no enrichment of 5hmC in the zebrafish during embryogenesis (36). In addition to these βGT-based protocols, 5hmC enrichment has also been carried out through antibody-based purification of modified forms of 5hmC (anti-cytosine 5-methylenesulphonate; CMS) (30) as well as techniques allowing potential single base resolution sequencing through either oxidative bisulphite sequencing (oxBS-seq) or TET assisted bisulphite sequencing (TAB-seq) (33,35). Although the single base resolution strategies have the potential to yield superior levels of information regarding genome-wide 5hmC distribution, the relative sequencing costs required to provide sufficient depth to cover the entire genome and accurately map the low amounts of 5hmC makes such techniques less cost effective than many of the alternative affinity-based methods presently available.

With this in mind, we set out to comprehensively test three affordable and widely used affinity-based techniques for 5hmC enrichment (antibody (hmeDIP); chemical capture (hMeSeal) and affinity purification (JBP-1 purification)) on two different mouse tissues, which vary in their global levels of the 5hmC modification (whole brain = high levels at 0.7% of dG, liver = intermediate/low levels at 0.07% of dG) (15,37). In contrast, 5mC levels (4.5% of dG) are comparable between the two tissues (37). We have compared the distributions of the enriched fragments on high-density 2.1 million probe tiled microarrays (which due to the total number and density of probes mirror published genome-wide sequencing datasets closely; see Supplementary Figure S1), allowing us to compare the 5hmc patterns generated by the various affinity-based enrichment techniques in a cost-effective manner without any loss of data integrity. Following these microarrays, we carry out independent validation of our results by both standard quantitative PCR (qPCR) as well as by a hydroxymethylation sensitive restriction enzyme PCR-based strategy (gRES-qPCR) which allows us to quantify absolute levels of DNA modification at single CpG dinucleotides. This technique has been previously used to successfully validate both HmeDIP based (15,25) and oxBS-seq derived (35) 5hmC datasets. Additionally, we also compare our data to already published 5hmC microarray-based datasets (25,26). From our analysis, we find that the JBP-1 affinity-based technique does not sufficiently enrich for 5hmC-containing DNA to allow for accurate mapping of the modification in the genomes of tissues analysed. In contrast, both hmeDIP and hMeSeal generated similar patterns of distribution on a probe by probe as well as the genic level, although higher levels of background noise were observed with hmeDIP. In addition, both of these enrichment techniques result in similar associations with chromatin modifications typically found at enhancers and gene bodies, while the JBP-1 enriched material showed less obvious relationships. Focusing on the hmeDIP and hMeSeal techniques, we define a small number of regions which display significant variance between these two datasets revealing that the antibody is not only biased towards CA repeats and Line1 elements as previously reported (28,31), but also recognizes a full spectrum of simple repeats. Finally, these regions of variance appear to be most pronounced across imprinted regions in our array set such as the *H19/Igfr2* and the *Tsix/Xist* loci, which are correspondingly enriched for simple repeats. As such, although both techniques accurately report the hydroxymethylome patterns in mouse tissues, we conclude that the hMeSeal chemical capture-based technique is currently the optimal affinity-based enrichment technique that is widely available for laboratories interested in the study of 5hmC.

## MATERIALS AND METHODS

### Mouse strains

Adult male C57BL/6 mice were sacrificed and livers taken for subsequent DNA extraction. DNA was extracted from small pieces of liver following digestion with proteinase K prior to phenol chloroform. During analysis, datasets were also compared to B6CH3HF1 hybrid mice (GSE40540).

### Purification of 5hmC and 5mC enriched DNA fragments

Prior to purification, genomic DNA was extracted from frozen (−80°C), ground-up livers and fragmented to an average of 500 bp for hmeDIP protocols and 300 bp for JBP-1 and hMeSeal protocols (Bioruptor, Diagenode). For an overview of the three techniques see Figure 1a.

#### Antibody (hmeDIP)

Genomic DNA was sonicated (Bioruptor, Diagenode) to produce DNA fragments ranging in size from 200 to 1000 bp, with a mean fragment size of around 300 bp. A total of 4 µg of fragmented DNA was immunoprecipitated for 3 h at 4°C with 2.5 µl of a rabbit polyclonal antibody against 5hmC (Active motif, cat#39769) in a final volume of 500 µl IP buffer (10 mM sodium phosphate (pH 7.0), 140 mM NaCl, 0.05% Triton X-100). This mixture was incubated with 60 µl of magnetic M-280 protein G Dynabeads (Invitrogen #100-03D) for 2 h prior to washing all unbound fragments three times with 1 ml IP buffer. Washed beads were then incubated with pK for 2 h at 50°C. Immunoprecipitated DNA fragments were then purified by passing through DNA purification columns (Quiagen) and eluting into Tris-EDTA buffer pH 8.0 (TE).

#### Chemical capture (hMeSeal)

Chemical capture (hMeSeal) methods were developed in the He lab at the Univeristy of Chicago (32) and marketed under the name Hydroxymethyl Collector Kit by Active Motif (cat# 55013). Following sonication of the DNA to a mean size of 300 bp, 1 µg of fragmented DNA was glucosylated through incubation with modified dUTP containing an azide glucose group with 20 U βGT enzyme for 1 h at 37°C. Following glucosylation, the modified glucose group was then biotinylated through incorporation of biotin conjugation solution for 1 h further at 37°C. Fragments of DNA containing modified biotin-azide-glucose-5hmC were then purified through binding to magnetic streptavidin beads following five washes with a wash buffer and a final wash in elution buffer for 30 min at room temperature. Purified DNA was then passed through DNA purification columns and eluted in elution buffer prior to qPCR and microarray hybridization. All chemicals described were supplied with Hydroxymethyl Collector Kit by Active Motif (cat# 55013). Please refer to manufacturers’ protocol for more information. Following the work carried out in this study, we found some long-term stability issues with latter batches of the active motif βGT enzyme. As such, we suggest substituting 20 U per reaction of the active motif T4-phage βGT enzyme for 30 U/rxn of the T4-phage βGT enzyme supplied from New England Biolabs (NEB, Cat# M0357L).

#### JBP-1 protein affinity

JBP-1 affinity pull-down technology was developed in the Klungland Lab at the Oslo University Hospital Rikshospitalet, Norway, and is marketed by Zymo research under the name Quest 5hmC DNA enrichment kit (cat#D5421). In short, Genomic DNA was sonicated (Bioruptor, Diagenode) to produce DNA fragments ranging in size from 200 to 1000 bp, with a mean fragment size of around 300 bp. Following the manufacturers protocols, 1 µg of fragmented DNA was then glucosylated through incubation with glucose-modified dUTP and 4 U βGT enzyme for 1 h at 37°C. Following glucosylation, the DNA was incubated with magnetic JBP capture beads containing the JBP-1 protein for 2 h at room temperature. Beads were then washed in wash buffer before eluting with the provided elution buffer. All chemicals described were supplied with name Quest 5hmC DNA enrichment kit supplied by Zymo research (cat#D5421). Please refer to manufacturers’ protocol for more information.

### Microarray hybridization

Following purification, material for validation was set aside and the remaining samples prepared for microarray analysis by whole genome amplification using WGA2:GenomePlex Complete Whole Genome Kit (Sigma). Amplified material was then labelled and hybridized to 2.1 M whole genome mouse tiling array set 2 of 4 (Roche Nimblegen), which covers a proportion of chromosomes 4 and 9 and all of chromosomes 5,6,7 and 8.

### Processing of Nimblegen tiled microarrays

Nimblegen 2.1 M deluxe mouse tiled arrays (mm9 build) contain 2 171 066 unique probes of 50–70 bp in length spanning 6777 unique genes. Signals for each probe of the 5hmC-enriched samples (Cy5 labelled) were compared to input samples (Cy3 labelled) to generate log2 (IP/Input) scores (fold changes). These log2 scores were then normalized first by Loess normalization and then by scale normalization using the Limma package in R/Bioconductor (38). For all samples, each probe was then mapped to one of five regions of the genome based on Refseq gene annotations: promoter core = TSS +100 bp to −100 bp, promoter proximal = TSS + 1 kb to +100 bp, promoter distal = TSS +2 kb to +1 kb, intra-genic = gene body or inter-genic = not associated with an aforementioned region (Figure 1e). Due to the highly reproducible nature of the patterns, average probe values were calculated for biological replicates for both brain and liver samples.

### Bioinformatic analysis

Genome-wide analysis of the datasets was carried out using R/Bioconductor or the Wellcome trust Centre for Cell Biology Galaxy server. Peaks representing probes that were enriched in 5hmC were defined in a similar fashion to those outlined in (26), but with the criteria changed so that at least three out of four probes had to reach the 95th percentile threshold. Peaks were mapped to one of five unique regions of the genome through direct overlap. Non-uniquely mapping peak probes (i.e. mapping to a promoter which overlaps a genic region of a second gene) were excluded from the analysis. Peak probes mapping to CpG islands were mapped in a similar way.

Average levels of 5hmC over genes were calculated by taking the average of all probes mapping to a particular gene (log2 scores) and then dividing by the length of the gene in base pair.

To calculate the probe differences in the 5hmC patterns generated between the hmeDIP and hMeSeal datasets, average 5hmC values were first calculated for biological replicates and the hmeDIP values subtracted from the hMeSeal log2 scores (frequently referred to as enrichment bias in the antibody relative to the chemical capture or Δ5hmC hmeDIP versus hMeSeal). Probes seen to differ greater than 1.5-fold over a window of at least three probes were selected for further analysis (Figure 3a).

Clustering of samples based on their DNA modification samples was calculated from 500 000 random probes (24.3% total unique probes on array). Dendrogram plots were carried out using R and distances calculated through both Euclidian and Ward methods. Scatter plots of 500,000 random probes or total genic 5hmC levels were drawn using the plot and smoothscatter functions. Kernel density plots were plotted using the density function. Boxplots were drawn using the function boxplot.

CpG calculations were carried out on the Wellcome trust Centre for Cell Biology Galaxy server using the ‘CpG calculation’ tool. Average patterns of 5hmC across genes were carried out using the tool ‘sliding window over length normalised regions of interest’. In short, this function takes a set of genomic coordinates (gene start + 25% upstream and stop + 25% downstream) and calculates the patterns of 5hmC from the supplied genome-wide data file based on the % length of each gene. Average signals were drawn for biological replicates.

### Bioinformatic analysis of ENCODE/LICR histone modification datasets

Genome-wide ChIP-seq data perfomed by the Ren lan, UCSC as part of the ENCODE project, was downloaded from the UCSC genome browser database in accordance with published guidelines (39). All datasets were analysed following the arbitrary 9 month ‘moratorium’ on the publication that expired on December 2012. Following antibody enrichment, samples were sequenced on Illumina Genome Analyzer II, Genome Analyzer IIx and HiSeq 2000 platforms for 36 cycles. Image analysis, base calling and alignment to the mouse genome version NCBI37/mm9 were performed using Illumina's RTA and Genome Analyzer Pipeline software. Alignment to the mouse genome was performed using ELAND or Bowtie with a seed length of 25 and allowing up to two mismatches. Only the sequences that mapped to one location were used for further analysis. Patterns of histone modifications were subsequently generated and peaks of enrichment were calculated, both of which are freely available on the USCS browser under the tab ‘LICR Histone Mods’.

For our analysis, we compared the peaks of 5hmC enrichment to the reported peaks of histone modification. Data was first restricted to the regions present on the mouse 2.1 M high-density tiled microarray 2 of 4 (Roche Nimblegen). Following this, regions of the genome which contained a peak of only one histone modification (i.e. only H3K4me3 and not also H3K27me3 for example”) were defined by subtracting the coordinates of the peak sets for each mark. Plots of overlap were generated as a proportion of the total number of these unique histone peaks which also overlap by at least 1 bp with a peak of 5hmC.

Sliding window analysis was carried out using the same procedure outlined above to calculate average 5hmC profiles across gene bodies (through the use of the Wellcome trust Centre for Cell Biology Galaxy server ‘sliding window over length normalised regions of interest’ tool). Midpoints of histone peaks were calculated and regions expanded out + and −5 kb. 5hmC patterns from our arrays were tested across these 10 kb windows.

### Definition of simple tandem repeats and repeat elements

List of simple tandem repeats and repeat elements were generated by using the tandem repeat finder program supplied by the Boston University and the repeat masker program developed by the Institute for systems biology, Seattle. Both sets of data were available through the UCSC genome browser at http://genome.ucsc.edu.

### Glucosylation-mediated restriction enzyme sensitive qPCR (gRES-qPCR)

The EpiMark kit (NEB) was used to quantify relative levels of 5hmC and 5mC at select loci in mouse brain and liver DNA. All data were scaled at each locus so that the total per cent of marks = 100 and as such only relative and not absolute levels of each mark to be calculated. For the full protocol see the manufacturer’s instructions. Typically, 10 µg of genomic DNA was taken and half treated with T4-phage βGT for 12–16 h at 37°C. Both the βGT treated and untreated samples were then divided into three PCR tubes and digested with either MspI, HpaII or left uncut for a further 12–16 h at 37°C. Samples were proteinase K treated for 10 min at 40°C prior to dilution to 100 µl final volume in H_2_0 and heating to 95°C for 5 min. qPCR was carried out on 5 µl (∼0.8 µg DNA) of each sample on a Roche LightCycler 480 PCR machine. Relative enrichments of the modifications were then calculated following formulae provided by NEB.

### Data access

Data available on the Gene Expression Omnibus (GEO) under accession number GSE51577. Published 5hmC and 5mC microarray datasets studied in this work can be found in GEO under the super-series GSE40540. HmeDIP-seq datasets generated by the Shen lab can be found in GEO under the super-series GSE42250. ChIP-seq datasets generated by the Ren lab as part of the ENCODE project are accessible via the UCSC genome browser.

## RESULTS

### High-density analysis of liver 5-hydroxymethylome patterns enriched by antibody, chemical capture and affinity-based methods

The number of potential tools for the enrichment of DNA modifications is increasing, with recent studies mapping even the low abundant 5fC and 5Cac modifications in mouse DNA (13,14). Previous work has been carried out to evaluate both affinity and antibody-based methods of 5mC enrichment reporting discrete technique dependant differences (40). We set out to comparatively test the 5hmC enrichment properties of the widely used immunoprecipitation-based purification method (hmeDIP), with respect to two recently reported antibody independent methods, namely selective chemical labelling (hMeSeal) (32) and JBP-1 affinity purification (34). An overview of the techniques and chemistry involved in these three methods is outlined in Figure 1a. In brief, hmeDIP is based on immunoprecipitation of DNA fragments with antibodies highly specific for the 5hmC modification. In contrast, both the hMeSeal chemical capture and JBP-1 affinity capture techniques rely on several steps of chemical modification prior to purification, primarily involving the complete glucosylation of 5hmC modified cytosines using the T4 bacteriophage enzyme βGT. In the case of hMeSeal, the newly glucosylated cytosines are then biotinylated permitting high-affinity streptavidin purification. The JBP-based purification techniques rely on the affinity of the JBP-1 protein for glucosylated DNA, which in turn is purified through the binding to magnetic beads.

We initially tested the relative enrichment properties of these three techniques over a cohort of loci which we previously identified to be either enriched or depleted in the 5hmC modification in the mouse liver (26) by quantitative PCR (qPCR) (Figure 1b). From this analysis, it was apparent that both the hmeDIP and hMeSeal approaches were specifically enriching for 5hmC marked regions of DNA, while the results of the JBP-1 affinity purifications, although showing a similar trend, were weaker. This is likely due to the fact that the amount of DNA recovered following the use of this approach was far lower than the hmeDIP and hMeSeal techniques. For example, qPCRs carried out on hmeDIP and hMeSeal enriched material typically reached their cycle threshold of amplification (Ct) between 30 and 40 cycles while JBP purified DNA fragments routinely took >50 cycles to reach the cycle threshold (*C*_t_) indicating that the amount of DNA present is extremely low (Supplementary Figure S2a). To ensure this was not due to an isolated issue with the JBP-1 protein preparation, we repeated the enrichment using a second batch of JBP-1 and achieved similarly high *C*_t_ values (Supplementary Figure S2). As such JBP-1-based techniques of purifying 5hmC enriched DNA resulted in low levels of enrichment relative to input DNA compared to hMeSeal and hmeDIP (Supplementary Figure S2b). The increased variability introduced by high cycle thresholds makes it difficult to reliably quantify levels of the modification using JBP-1. Interestingly, *C*_t_ values were also higher for the hMeSeal enriched DNA compared to those obtained with hmeDIP (Supplementary Figure S2a) highlighting that although relative enrichment values across loci are high for this approach, the absolute level of 5hmC enriched DNA returned is lower. Significantly, relative levels of 5hmC enrichment were similar for all three approaches irrespective of the absolute enrichment levels observed (Figure 1b).

Following this candidate-driven approach, we wished to test the ability for these techniques to enrich and therefore report on the patterns of 5hmC modification across a large proportion of the mouse genome. To do so, we carried out purification of 5hmC marked DNA in both mouse brain and liver tissues (*n* = 2) using the hmeDIP and hMeSeal techniques prior to hybridization on high-density tiled microarrays covering ∼25% of the mouse genome (chromosomes 4–9). Due to the low levels of 5hmC enrichment of 5hmC using the JBP-1 affinity-based method observed through qPCR (Figure 1b and Supplementary Figure S2), JBP-1 profiling was performed on a single brain sample as this tissue would contain the high levels of 5hmC required by JBP-1. In agreement with the qPCR results described earlier, hierarchical clustering of the microarray data reveals that the 5hmC patterns generated by the JBP-1 affinity approach clusters completely independently and is lowly correlated (Pearson’s correlation value <0.25) from those purified by hmeDIP or hMeSeal (Figure 1c). In contrast, the hmeDIP and hMeSeal datasets were highly correlated (mean Pearson’s correlation values of >0.79 for brain and 0.71 for liver datasets; Figure 1c), and clustered for each tissue. Furthermore, low levels of individual variation was noted between biological replicates of each technique (for example, Pearson’s correlation values of 0.87 between the brain 1 and brain 2 HmeDIP sets; Figure 1c) which agrees with results of recent work highlighting the reproducible nature of the 5-hydroxymethylome between individuals (25).

To gain an overview of the signal-to-noise ratio and periodicity of the data, we performed autocorrelation analysis of the tiling microarray data for hme-DIP, hMeSeal and JBP-1 binding in a single brain sample (Figure 1d). For comparison, a randomized data set was generated from the hMeSeal data. Probe values for both the hMeSeal and hme-DIP data were strongly correlated with that of neighbouring probes to ∼2 kb, and show continued association across the 10-kb region analysed. Interestingly, although the pattern of autocorrelation was highly similar for both hmeDIP and hMeSeal, the latter showed consistently higher autocorrelation values reflecting a higher signal-to-noise ratio to that of hmeDIP. Relative to the hmeDIP and hMeSeal data, the JBP-1 data showed vastly reduced correlation between probes at all distances assayed, and closely resembled the autocorrelation profile obtained with random data (which was also apparent from the microarray profiles; Supplementary Figure S3), reflecting a markedly lower signal-to-noise ratio.

It has been widely reported that 5hmC modified DNA is typically found in the bodies of actively transcribing genes (17,18,26,28,29,32,41) and enhancer elements (13,41,42) and that overall 5hmC patterns are highly tissue specific (15). Accordingly, hierarchical clustering of the high-density tiled microarray datasets reveals that the 5hmC patterns vary more between tissues than between techniques of enrichment (Figure 1c), reinforcing the overall similarity of the 5hmC datasets generated by the hmeDIP and hMeSeal (but not JBP-1 affinity) techniques. Subsequent visualization of the relative 5hmC patterns across tissue-specific genes such as the liver-specific gene *Cyp2b10* reveals the ability of the hmeDIP and HmeSeal techniques to reflect the tissue-specific 5hmC patterns (Figure 1e). Furthermore, both techniques display highly reproducible patterns of 5hmC enrichment between individuals as seen by the similar profiles in the biological replicates of each technique (Figure 1e). To independently validate these findings, we carried out glucosylation-mediated restriction enzyme sensitive qPCR (‘gRES-qPCR’, see ‘Materials and Methods’ section) over regions containing a single *Msp*I cut site. In short, this is based on the relative ability for the restriction enzymes HpaII and MspI to cut 5mC, 5hmC and non-modified CpG dinucleotides in the sequence CCGG. When glucosylated, the MspI enzyme can no longer recognize the modified CpG and the relative ability to subsequently amplify by qPCR is representative of the cut frequency and hence modification status (see ‘Materials and Methods’ section). The gRES-qPCRs carried out over loci at the *Cyp2b10* gene strongly validate both the hmeDIP and hMeSeal techniques while once more highlighting the non-specific nature of JBP-1 purification techniques (Figure 1e and Supplementary Figure S3).

To fully characterize the 5hmC patterns generated by each technique, we generated datasets of probes residing in regions enriched in 5hmC (peak probes: see ‘Materials and Methods’ section). This analysis reveals that globally more peak probes are defined in the hMeSeal generated libraries (e.g. 161 142 peaks in the average profiles from the two brain samples) than for the hmeDIP counterpart (130 560 peaks in brain; Supplementary Figure S4) while far fewer peaks were identified following JBP enrichment (22 561 brain peaks). The mapping of these peaks to one of five unique regions genome was carried out to test whether the distribution of 5hmC enrichment differed between the techniques. In agreement with published studies (18,26,28,29,32,33), the majority of 5hmC enriched peak probes from the hmeDIP and hMeSeal datasets mapped to genic loci (‘Intra-genic’, Figure 1f) while a smaller fraction map between genes (‘Inter-genic’, Figure 1f) or to regions at or around promoters (‘promoter distal’; TSS +2 kb to +1 kb, ‘promoter proximal’: TSS +1 kb to +100 bp and ‘promoter core’; TSS±100 bp, Figure 1f). In contrast, the JBP-1 enriched peaks mapped largely to inter-genic loci (Figure 1f). Between the hmeDIP and hMeSeal peak sets, there was a notable difference in the enrichment of 5hmC peaks over the promoter proximal regions (liver: +1.03%, brain: +0.8% total probe enrichment in hMeSeal versus hmeDIP datasets) in the libraries purified by hMeSeal. Taken together, both hmeDIP and hMeSeal based techniques are ultimately capable of reporting similar hydroxymethylome patterns.

### Antibody-based enrichment of 5hmC DNA reveals a slight CpG sequence bias

As previous studies have reported a CpG bias associated with the antibody-based purification techniques (31), we set out to test this within our dataset in comparison to the hMeSeal protocol. For both brain and liver samples, we observe a shift in the number CpGs found across enriched DNA fragments between the two techniques (Supplementary Figure S5). This difference is most clearly observed in the 5hmC rich brain samples in comparison with the liver, which contains lower 5hmC levels globally. Consistent with previous reports, we find that in both tissues the antibody-based purification method enriches for fragments of DNA containing higher densities of CpG dinucleotides than the hMeSeal technique (mean CpG observed/expected 0.284 and 0.228 for brain and liver hmeDIP respectively, 0.22 and 0.198 for brain and liver hMeSeal; Supplementary Figure S5). As such, the hMeSeal technique may be important in the study of 5hmC over regions of low CpG composition, whereas the antibody may be better suited to the study of hydroxymethylation at CpG islands and promoters with more intermediate levels of CpG density (ICPs). Extension of our analysis to investigate the number of 5hmC marked peak probes aligning to annotated CpG islands in our datasets reflects this. CpG islands are typically devoid of any form of cytosine modification and we find that ultimately all three affinity-based techniques are unable to purify such loci, with the total number of peak probes mapping to a CpG island <0.11% of total probes in either tissue by either technique (Figure 1g). There is however a larger proportion of promoter CGIs marked by 5hmC following JBP-based affinity enrichment; however, when taken together with the earlier observations (Figure 1c, d and f and Supplementary Figure S3), this is likely to be not significant due to background noise.

### Analysis of genic 5hmC patterns between hmeDIP and hMeSeal generated datasets

**Figure 2.**
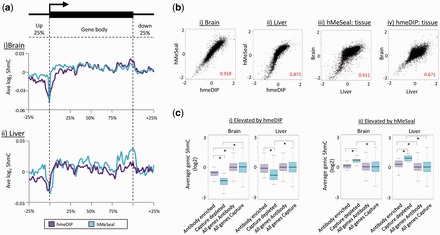
Genic patterns of 5hmC enrichment are similar following enrichment by hmeDIP or hMeSeal. (**a**) Average pattern of 5hmC across promoter and genic regions are highly similar following independent purification by either hmeDIP (purple) or hMeSeal (blue) in both the brain (i) and liver (ii). Average 5hmC values were calculated at positions relative to the full length of the gene with analysis extending 25% up and downstream. *Y*-axis plots average log2 scores taken from microarray datasets. (**b**) Scatter plots of average length adjusted genic levels of 5hmC between hmeDIP and hMeSeal in the brain (i) and liver (ii) tissues (each *n* = 2). Scatterplots are also shown to compare the relationships between the genic 5hmC levels across tissues by both hMeSeal (iii) and hmeDIP (iv). Pearson correlation values are shown in red in each plot. Data are plotted for average log2 scores from −2 to +2 for the 5hmC content of each gene following length adjustment. (**c**) Box plots of average length normalized 5hmC levels across genes exhibiting a strong relative enrichment following either antibody (i) or chemical capture (ii) purification compared to all genes on the array. Antibody purified; purple, chemical capture purified; blue. Genes enriched by hmeDIP versus hMeSeal are typically depleted in 5hmC with respect to all genes indicating higher levels of noise. Conversely, regions enriched by hMeSeal are genuinely enriched over the bulk of the genes on the array.*Denotes Willcox *P-*value scores <0.05.

As the majority of 5hmC enriched DNA is found within genic regions, we next focused our analysis on these regions. Plots of the average 5hmC patterns across all genes ±25% of their length present on the high-density tiled microarrays (*n* = 6777) reveal highly similar promoter and genic 5hmC patterns in both liver and brain (Figure 2a) following both hmeDIP and hMeSeal enrichment. Typically, this presents itself as a dramatic loss of 5hmC directly over the TSS with an elevated gene body 5hmC content which dips down again over the 3′-end. Although both hmeDIP and hMeSeal brain and liver datasets exhibit a clear drop off in the 5hmC signal directly around the TSS, levels of 5hmC are heightened over promoter proximal regions following chemical capture-based enrichment (which agree with our earlier observations regarding the distributions of peak probes, Figure 1f). In stark contrast, JBP1 enriched patterns are the direct opposite of those seen when enriched by hmeDIP or hMeSeal approaches, with a peak of 5hmC observed directly over the TSS (Supplementary Figure S6)—a result which is not supported by the field at present. Focussing on the hmeDIP and hMeSeal datasets, further analysis reveals that the average levels of 5hmC in each gene are highly correlated when comparing the two techniques (cor values of 0.918 and 0.872 for brain and liver respectively, Figure 2b, i and ii) while the levels of correlation are consistent between the two techniques when comparing values between the tissues (cor values of 0.611 and 0.671 for hMeSeal and hmeDIP, respectively, Figure 2b, iii and vi). Taken together, both techniques appear to be capable of ultimately generating similar 5hmC enriched datasets. Although the use of both techniques results in the generation of largely similar genome wide 5hmC patterns, we found that a small group of genes which were reproducibly differentially enriched in both biological replicates depending on the technique used to purify the fragments (brain = 203, liver = 44); Supplementary Figure S7a). Subsequent analysis revealed that many of the hmeDIP enriched genes contain lower levels of 5hmC than is found across the rest of the genome (antibody enriched/hMeSeal depleted genes: mean log2 −0.43 by hmeDIP, mean antibody 5hmC levels across genome: log2 +0.014 by hmeDIP) and that this elevation in the antibody enriched datasets relative to the chemical capture sets was in fact due to the lower values reported by hMeSeal over genuinely 5hmC-depleted loci (Figure 2c and Supplementary Figure S7b). Indeed, genome browser visualization of many of these hmeDIP enriched genes found that they clustered to large regions of the genome which were vastly depleted in the 5hmC modification (Supplementary Figure S7b), which would indicate that higher levels of noise are introduced by the antibody-based approach in such regions. In contrast, the small number of genes with elevated genic 5hmC in the hMeSeal dataset (brain *n* = 30, liver *n* = 18) appear to be genuinely enriched over general genomic 5hmC levels by both techniques (Figure 2c and Supplementary Figure S7c). The functional significance of the elevated genic 5hmC in these genes is however unknown as GO term analysis yielded no particular set of genes with overlapping functional pathways. Nevertheless, it appears that the hMeSeal can enrich for 5hmC with less noise compared with the antibody-based approach.

### Both hmeDIP and hMeSeal enriched DNA fragments associate with a specific subset of chromatin modifications and binding proteins

**Figure 3.**
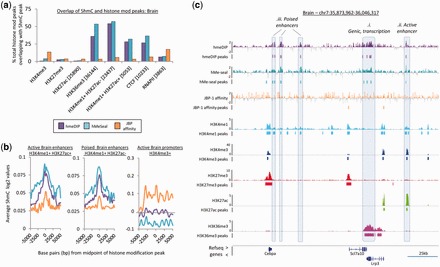
Regions of 5hmC enrichment are associated with select histone modifications. (**a**) Overlap between peaks of histone modifications/DNA binding proteins with peaks of 5hmC derived through the three purification techniques in the mouse brain. Plots show percentage of total histone modification peaks which overlap with a peak of 5hmC by at least 1 bp. Total number of histone modification peaks are shown below in square brackets. (**b**) Average 5hmC profiles over 10 kb windows at ‘poised’ and active enhancers compared with active promoters in the brain for each enrichment technique. (**c**) Genome browser visualization of 5hmC patterns (hmeDIP: purple, hMeSeal: teal, JBP-1 affinity: orange) overlap with select histone modifications (H3K4me1:light blue, H3K4me3: dark blue, H3K27me3: red, H3K27ac: green, H3K36me3: pink) in the mouse brain. Array data plotted on log2 scale while ChIP-seq the number of reads. Refseq genes are displayed below.

Following the analysis of the genome-wide patterns of 5hmC, we set out to relate these pattern to a selection of commonly studied histone modifications with the aim of better understanding (i) the relationships between the 5hmC modification and the chromatin landscape and (ii) differences in these relationships between enrichment strategies. To do so, we analysed publically available ENCODE (‘ENCODE/LICR histone modification’ tracks) genome-wide sequencing datasets generated by the Ren lab at the University of California, San Diego, for both mouse brain and liver tissue samples. In short, five histone modifications were selected: H3K4me3 (found at active promoters), H3K27me3 (found at bivalently marked promoters and transcriptionally silent gene bodies), H3K4me1 (found over poised enhancers as well as some promoters), H3K27ac (found over active promoters as well as active enhancers when marked by H3K4me1) and H3K36me3 (found over actively transcribing gene bodies). In addition to these histone modifications, we also investigated the relationships between 5hmC presence and the binding of CTCF (an insulator protein) and RNA polymerase II (indicative of active promoters). To study the overlap between the sequencing datasets, data were first restricted to the regions covered on the microarray and resulting Encode defined peaks compared for an overlap with 5hmC peaks. Co-localization of peaks was defined as overlapping peaks mapping to regions of single histone modification (i.e. regions containing only H3K4me3 and not H3K4me3 and H3K27me3) displayed as a percentage of the total number histone modification peaks (Figure 3a and Supplementary Figure S8a). In agreement with the general 5hmC patterns observed over genes (Figure 2a), modification patterns that are typically found over promoter regions (H3K4me3+, H3K27ac+ & RNAP II+) were depleted in their relation to the 5hmC mark by following both hmeDIP and hMeSeal enrichment, but not JBP affinity purification which instead exhibited moderate enrichment (Figure 3a–c and Supplementary Figure S8a). No discernible relationship was observed between the 5hmC modification and regions rich in the H3K27me3 mark following any method of 5hmC enrichment—which agrees with the notion that the 5hmC modification is largely euchromatic (17,28–30,32). In contrast, both the hmeDIP and hMeSeal enriched datasets were found over a large number of H3K36me3+ve peaks (36% and 53.4% of total H3K36me3 peaks in the brain and 39% and 64% total H3K36me3 peaks in the liver for hmeDIP and hMeSeal, respectively; Figure 3a and c and Supplementary Figure S8a) which relates to the well-cited relationship with actively transcribing genes as well as the global distribution of the 5hmC marks outlined in Figure 1f. 5hmC has been previously shown to be enriched at enhancer elements which may in turn be functionally important for the expression of nearby genes (13,41–44). Accordingly, we observe high levels of overlap between peaks of 5hmC at predicted active enhancers marked by H3K4me1+/H3K27ac+ (28% and 32% of total active enhancers in the brain and 43% and 46% in the liver for hmeDIP and hMeSeal, respectively) as well as poised enhancers marked by H3K4me1+/H3K27ac− (54% and 57% of total poised enhancers in the brain and 62% and 84% in the liver for hmeDIP and hMeSeal, respectively; Figure 3a–c and Supplementary Figure S8a). Once more JBP-based affinity derived patterns of 5hmC do not show similar relationships with the H3K36me3, H3K4me1 and H3K27ac marks. Finally, hmeDIP and hMeSeal (but not JBP affinity) derived 5hmC peaks are seen to overlap with a proportion of regions which are bound by the insulator protein CTCF, which may relate 5hmC distribution to the regulation of higher order chromatin compaction. Taken together, these data suggest that the 5hmC fragments generated by either hmeDIP and hMeSeal purification (but not those from JBP-1 affinity) associate with similar distributions of histone modification, with a general elevation in overlap seen following hMeSeal-based methods (i.e. increased H3K36me3+, enhancer element and CTCF occupancy in the chemical capture-based 5hmC dataset relative to antibody-based datasets; Figure 3 and Supplementary Figure S8).

### Regions of differential enrichment between hmeDIP and hMeSeal reveal an antibody bias towards simple tandem repeats

**Figure 4.**
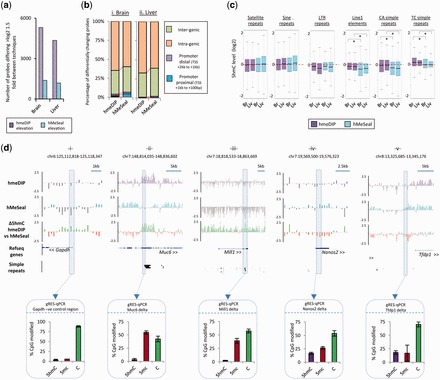
A small number of loci show high levels of technique specific enrichment bias. (**a**) Plot of the total number of probes on the microarrays which exhibit >log2 1.5-fold differences in 5hmC levels across both brain and liver tissues following either hmeDIP (purple) or hMeSeal (blue). (**b**) Genomic distributions of the differential probes shown in Figure (a) in both brain (i) and liver (ii) mapped to the same regions outlined in Figure 1e. Values are shown as percentages of all probes (%). Red lines highlight the relative changes in the genomic distribution. (**c**) Box plots of average 5hmC levels (log2 values) across a host of repetitive elements and simple tandem repeats reveal some antibody specific bias. 5hmC levels in datasets generated by hmeDIP (purple) or hMeSeal (blue) are plotted both for brain (Br) and liver (Liv). hmeDIP bias is seen at line1 elements as well as at CA and TC simple repeats.*Denotes Willcox *P*-value scores <0.05. (**d**) Examples of strong regions of technique specific enrichment bias. Microarray patterns of 5hmC enriched by hmeDIP (purple) and hMeSeal (blue) are shown on log2 scales from +2.5 to −2.5. Regions of depletion are marked as grey bars for each technique. Genomic coordinates are given above for mouse mm9 build. Relative changes in the 5hmC signal between the two techniques are also displayed with green bars indicating hmeDIP bias and red bars hMeSeal bias. Gene structures are given below with directionality denoted by arrows. Regions rich in simple repeats are displayed as solid black bars. Scale bars are shown above for reference. Boxed areas represent regions validated independently by gRES-qPCR. Boxed bar plots represent the percentage of each modification at a single CpG in the sequence CCGG following normalization (purple; 5hmC, red; 5mC, green; C). Error bars display the standard error of the biological replicates.

Although the general 5hmC patterns generated following antibody or hMeSeal-based purification (Figures 1c, e and f and 2a), a small number of regions were found to contain high levels of high variance between the two datasets. To analyse these regions, we selected probes residing in large regions (>200 bp) containing high levels of change in either the antibody generated dataset or the chemical capture dataset (Figure 4a, see ‘Materials and Methods’ section). It is important to note that these peak sets represent a very low proportion of the total probe count (brain; 6674 probes or ∼0.3% of total, liver; 5529 probes or ∼0.25% of total) once more indicating how reproducible the majority of the data is between the two techniques. Nevertheless, these regions are consistently variable between the techniques in both biological replicates, containing >1.5 fold (log_2_) change between the corresponding probe values in each dataset over a window covering at least three probes. Relative to hMeSeal, the hmeDIP libraries contained a greater number of differentially enriched probe regions—which is likely to represent the higher levels of background noise in these datasets (Figure 4a).These variable regions differed in their genic distribution from that of the majority of enriched probes (Figures 1f and 4b); mapping largely to non-genic regions (‘intra-genic’; Figure 4b). In agreement with earlier results (Figures 1f and 2a), the chemical capture-based enrichment dataset was also found to contain a greater proportion of specific peaks at regions directly upstream of the TSS (‘promoter proximal’; Figure 4b).

Previous work has indicated that antibody methods of 5hmC enrichment are biased towards CA simple repeats in mouse ES cell data (31). Therefore, we set out to test whether or not the regions of high variance between the two techniques occurred over any particular class of repetitive sequences. Analysis of the average 5hmC levels in the probes associated with a specific family of repetitive elements and simple tandem repeats reveals that hmeDIP approaches enrich for 5hmC over several subclasses of repeat, mainly those belonging to Line1 elements and simple repeats (all Willcox *P*-values <0.05, Figure 4c). In contrast, the levels of 5hmC present at the Line1 elements and simple repeats following hMeSeal enrichment are significantly lower (all Willcox *P*-values <0.05, Figure 4c). In all cases (with the exception of the TC simple repeats), the levels of 5hmC at these loci are lower than that observed for genic probes (Supplementary Figure S9) indicating that although enriched by the antibody, these repetitive elements have lower levels of 5hmC compared to gene bodies.

Following this result, we find that many of the strongest regions of variance showing antibody-specific enrichment are indeed found to correspond not only to simple tandem repeats but to a whole spectrum of simple repeats of varying length (Figure 4d, ii and iii). However, regions of hMeSeal elevation cannot be explained in such a way but may simply represent clearer enrichments than the antibody (Figure 3d, iv and v and Supplementary Figure S7c). To test which of the two techniques was reporting on the true 5hmC patterns over these loci, we carried out gRES-qPCR at variable regions containing a single *Msp*I sites. As this technique is independent of the purification steps required for hmeDIP and hMeSeal, it provided useful validation of the levels of modification present over a single CpG site at a specific locus. Accordingly, a 5hmC negative control region within the *Gapdh* promoter CpG island shows low levels of modification present at this site following gRES-qPCR (Figure 4d, i). The loci containing hmeDIP elevated 5hmC signals at regions containing simple repeats were found in fact to contain low levels of 5hmC by gRES-qPCR (*Muc6*: 3.13% & *Mill1*: 2.51% of CpGs are found to be marked by 5hmC; Figure 4d, ii and iii) indicating that these enrichments are likely an artefact of the antibody purification technique. Interestingly regions of hMeSeal elevated signal were seen to be genuinely enriched in the mark (*Nanos2*: 19.8% & *Tfdp1*: 18.1% of CpGs are found to be marked by 5hmC; Figure 4d, iv and v). Although CT(n) simple repeats have previously been observed to be enriched in the antibody-based technique and absent in the chemical capture (31), here we show that the nature of the repeat-based bias associated with the 5hmC antibody is far greater than previously noted.

### The Imprinted loci at *Tsix/Xist* and *H19/Igf2r* reveal striking regions of technique dependent 5hmC variance

**Figure 5.**
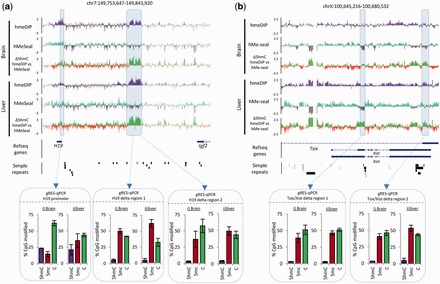
Imprinted loci at the *H19/Igf2r* (**a**) and *Tsix/Xist* (**b**) genes contain strong regions of technique dependant enrichment bias. Microarray data is plotted as describe earlier for Figure 3d. Enrichment by hmeDIP = purple track, hMeSeal= blue track, regions of depletion = grey in both tracks, hmeDIP bias = green track, hMeSeal bias = red track. Genes are displayed below as blue bars and simple repeats as black bars. The simple repeat highlighted in red refers to a region of TC tandem repeats. Boxed areas represent regions validated independently by gRES-qPCR (see description in Figure 4d).

In the course of our global analysis, we found that two of the most widely studied imprinted loci, *H19/Igfr2* and *Tsix/Xist* were also highly variable in their 5hmC patterns in a technique dependant manner (Figure 5). The epigenetic landscape at imprinted loci is thought to be particularly important for the maintenance of particular expression states and it is therefore crucial that quantitative DNA modification levels can be measured. Not only do the 5hmC patterns differ in the datasets reported here, but these same regions also differ when we analyse previously published datasets from a study investigating the changes to mouse liver 5hmC following exposure to the non-genotoxic carcinogen, phenobarbital (25) (Supplementary Figure S10). For the *Tsix/Xist* locus, these differences can be attributed to the aforementioned alignment with regions of simple repeats (Figure 5b). Although the large region of hmeDIP specific 5hmC enrichment between the *H19* and *Igf2r* genes did not align to many simple repeats, there is an enrichment of TC simple repeats towards the 3′-end of the locus (Figure 5a, simple repeats; red box) (Supplementary Figure S11). gRES-qPCR at these loci reveals that hMeSeal enrichment appears to best represent the true hydroxymethylome over these repetitive regions (Figure 5). Interestingly, the strong region of hmeDIP specific enrichment at the *H19/Igf2r* locus is also seen to be strongly enriched for the 5mC modification following immunoprecipitation with antibodies specific for the 5mC mark (methyl-DNA immunoprecipitation; MeDIP, Supplementary Figure S12), which can be independently validated as 5mC enriched by gRES-qPCR (between 37–49% and 46–62% 5mC at four *MspI* sites at these loci in brain and liver, respectively; Figure 5). As dot blot analysis has previously shown that the 5hmC antibody harbours no specificity towards 5mC modified DNA sequences, and vice versa (26), it is unlikely that the 5hmC antibody is cross reacting to the 5mC mark. Therefore, this region may exhibit a strong 5hmC signal following hmeDIP enrichment due to the high levels of repetitive sequence at this locus.

## DISCUSSION

Since its ‘re-discovery’ in 2009, research involving 5-hydroxymethylated DNA has increased at an incredible rate (for a review see (6)). The field has been driven by a marked increase in the number of potential tools for analysing the mark, each with its particular set of advantages and disadvantages (for a recent review see (5)). With our knowledge of the 5hmC modification expanding rapidly, comparison of 5hmC enrichment techniques is timely. In this study, we compared three widely used affinity-based techniques for 5hmC purification (antibody-based, chemical capture-based and JBP-1 affinity-based techniques) across mouse tissues containing either high (whole brain) or moderate (liver) levels of the modification. Generally, it was noted that both the antibody (hmeDIP) and chemical capture (hMeSeal)-based techniques were very similar in their genome-wide enrichments of the 5hmC modification (enriched over genic loci, depleted over TSSs) and exhibited high levels of tissue specificity as well as low levels of inter-individual variation (Figure 1b–d). In contrast, the 5hmC patterns generated through affinity-based enrichment with the trypanosome JBP-1 protein differed significantly (Figure 1b–d and Supplementary Figures S2, S3 and S6). In agreement with previous reports, there was a slight bias towards CpG-rich regions of the genome in the hmeDIP-based technique (31) (Supplementary Figures S5); however, these differences did not extend to CpG islands, which remain largely modification free in the tissues examined (Figure 1g).

Analysis of the chromatin modifications which are associated with peaks of 5hmC following enrichment by the three affinity-based purification techniques once more revealed the similar nature of the 5hmC landscapes generated following hmeDIP and hMeSeal. In the hmeDIP and hMeSeal datasets, 5hmC was seen to overlap with regions marked by H3K36me3 (a genic mark of active transcription) as well as both poised (H3K4me1+/H3K27ac−) and active (H3K4me1+/H3K27ac+) enhancers and CTCF sites, a result previously reported by others (41,45). In all cases, the hMeSeal-based enrichment showed elevated levels of enrichments between the DNA modification and the histone marks which may represent a superior method of 5hmC enrichment. 5hmC patterns generated following JBP-1 affinity-based methods failed to relate these same chromatin states, once more revealing the shortcoming of using such an approach to study the 5hmC modification.

Although the majority of probes on the arrays were similar in their enrichment levels following purification by the two techniques, a small subset (between 0.25% and 0.31% of total probes) were found to vary by greater than log2 1.5-fold (Figure 3a). The bulk of these could be attributed to an increase in the overall purification levels at particular loci, which did not affect the general distributions of 5hmC enriched by the two methods (Figure 3d and Supplementary Figures S5 and S6), while a subset were dramatically enriched only in the antibody-based method. It was noted that many of these regions were overlapping or adjacent to genomic regions containing simple repeats and tandem repeats (Figure 3d). Additionally, large regions of hmeDIP specific enrichment were noted over the well-studied imprinted loci of *H19/Igf2r* and *Tsix/Xist* (Figure 4). Independent validation by glucosylation-mediated restriction enzyme sensitive qPCR (‘gRES-qPCR’) found little or no 5hmC modified CpG dinucleotides at these loci, which was in agreement with the hMeSeal-based datasets arguing that antibody-based enrichment techniques may harbor some bias over repetitive regions of the genome.

In conclusion, we find that both the antibody (hmeDIP) and chemical capture (hMeSeal)-based techniques, but not JBP-1 affinity-based methods, are accurate at reproducibly detecting genome-wide patterns of 5hmC across tissues containing both higher (whole brain) and lower (liver) levels of total 5hmC. Although the reasons for the relative failure of JBP-1-based affinity enrichment are unclear, several predictions can be made. The most likely is that the enrichment is working albeit at a low level as evidenced by the high cycle threshold values during qPCR compared to hmeDIP and hMeSeal. Alternatively, the JBP-1 protein may also exhibit bias to certain portions of the genome; primarily as the JBP-1 purified libraries were found to be enriched directly over the TSS, a result which differs significantly from our own hmeDIP and hMeSeal datasets as well as the many published hydroxymethylome patterns. Nevertheless, the complementarity of the hmeDIP and hMeSeal-based techniques provides researchers with independent methods of 5hmC enrichment and validation which is applicable to both genome-wide and locus-specific studies (25). While the reduced background noise and higher affinity towards particular chromatin states observed with chemical capture-based methods makes this a highly attractive approach for the analysis of genome-wide 5hmC patterns (Figure 1d), antibody-based methods typically result in a greater total return of enriched DNA (as indicated by higher cycle threshold values generated during qPCR, Supplementary Figure S1) which may be more suitable for attaining enough material for downstream analysis. Ultimately, both techniques are suitable for the interrogation of 5hmC patterns at the non-repetitive regions of the genome. One major drawback of all of these techniques is that the enriched DNA fragments are all relatively large in size (sonication shearing of DNA typically results in fragments 150 bp–1 kb). As such it is hard to accurately determine the modification status of a single CpG when starting with DNA fragments of this length. In future, it is possible that these three methods of 5hmC enrichment may be superseded by technologies which can map 5hmC at single base pair resolution in a cost-effective manner (33,35). Nevertheless, both hmeDIP and hMeSeal-based enrichment methods can provide a cost-effective and accurate representation of the 5hmC landscape.

## SUPPLEMENTARY DATA

Supplementary Data are available at NAR Online.

## FUNDING

Innovative Medicine Initiative Joint Undertaking (IMI JU) under grant agreement number [115001] (MARCAR project) in part. Funding for open access charge: University of Edinburgh via grants from the MRC and IMI.

*Conflict of interest statement*. None declared.

## Supplementary Material

Supplementary Data
