# Supplementary material for: Comparative analysis of affinity-based 5-hydroxymethylation enrichment techniques
Source: Nucleic Acids Res. 2013 Nov 7;41(22):e206. doi: 10.1093/nar/gkt1080 (PMC3905904; doi:10.1093/nar/gkt1080)
Supplement: Supplementary Data [file supp_41_22_e206__index.html]

Comparative analysis of affinity-based 5-hydroxymethylation enrichment techniques — Comparative analysis of affinity-based 5-hydroxymethylation enrichment techniques — Supplementary Data 

# Comparative analysis of affinity-based 5-hydroxymethylation enrichment techniques

## Supplementary Data

files

**Files in this Data Supplement:**

- Supplementary Data - pdf file
