# Supplementary material for: Comparative analysis of affinity-based 5-hydroxymethylation enrichment techniques
Source: Nucleic Acids Res. 2013 Nov 7;41(22):e206. doi: 10.1093/nar/gkt1080 (PMC3905904; doi:10.1093/nar/gkt1080)
Supplement: Supplementary Data [file supp_gkt1080_nar-02616-met-k-2013-File007.pdf]

Supplementary figures

s1

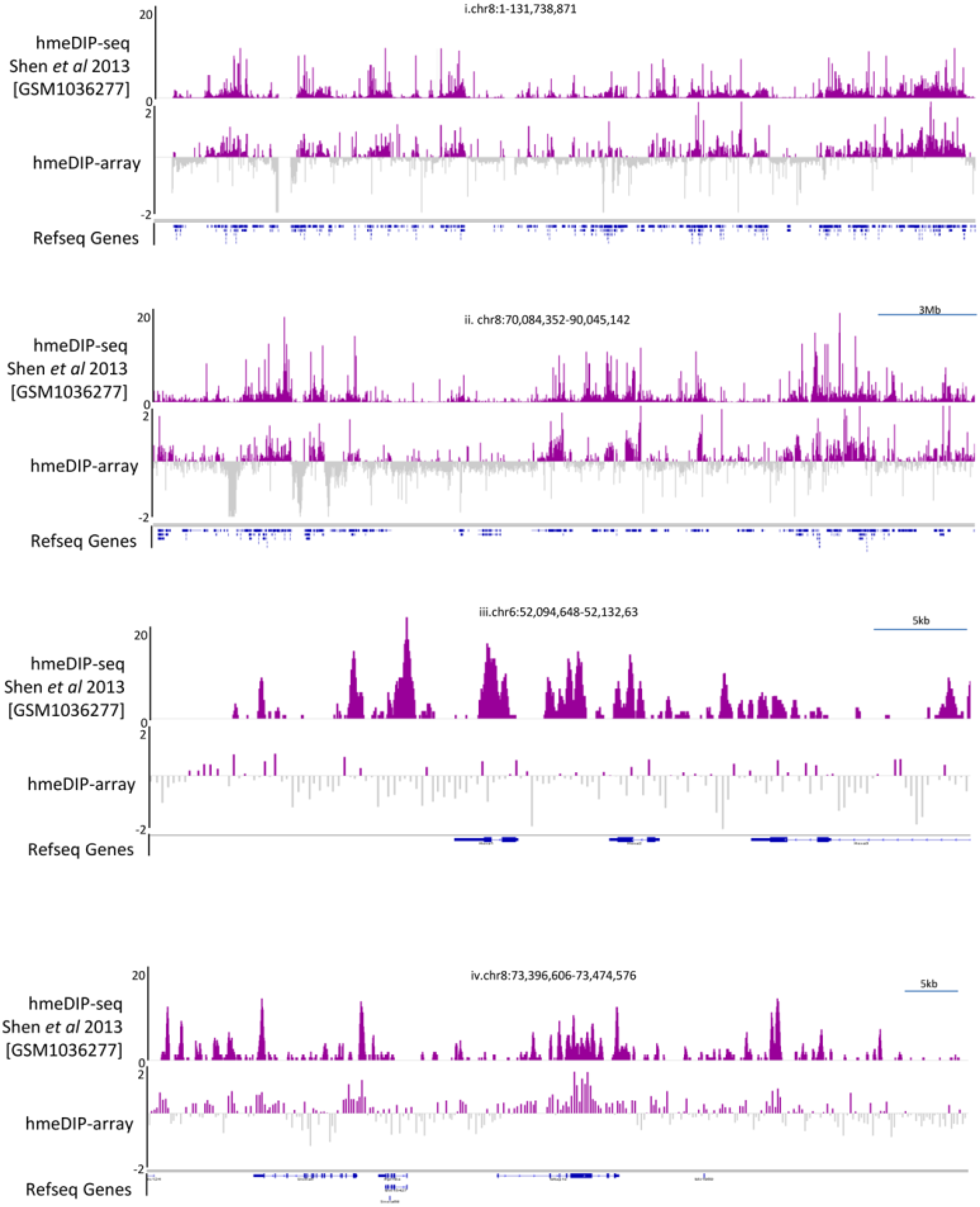

S1. Comparison of hmeDIP 5hmC profiles following whole genome sequencing approaches (Upper track – Shen et al 2013 hmeDIP-seq on mouse ES cells) to our high density tiled microarray profiling in the mouse brain. Although the patterns of 5hmC distribution appear largely similar at both the chromosomal level between ES cells and brain tissues (i & ii) genes which are unique in their transcriptional activities (such as the ES transcribed HoxA cluster) harbour distinct 5hmC profiles (iii). Many gene remain similarly expressed (such as at the microtubule associated gene Mtap1s – iv) and as such reveals similar patterns of 5hmC distribution. At such loci both hmeDIP-seq and hmeDIP-array report very similar patterns, indicating that using high resolution microarrays are viable is a cost effective method of accurately reporting on the distribution of 5hmC marked DNA. Microarray data is plotted on log2 scale from -2 to +2 whilst sequencing data plotted on the number of reads from 0 to 20. Refseq genes are annotated below (blue bars).Scale bars shown to the top right.

s2

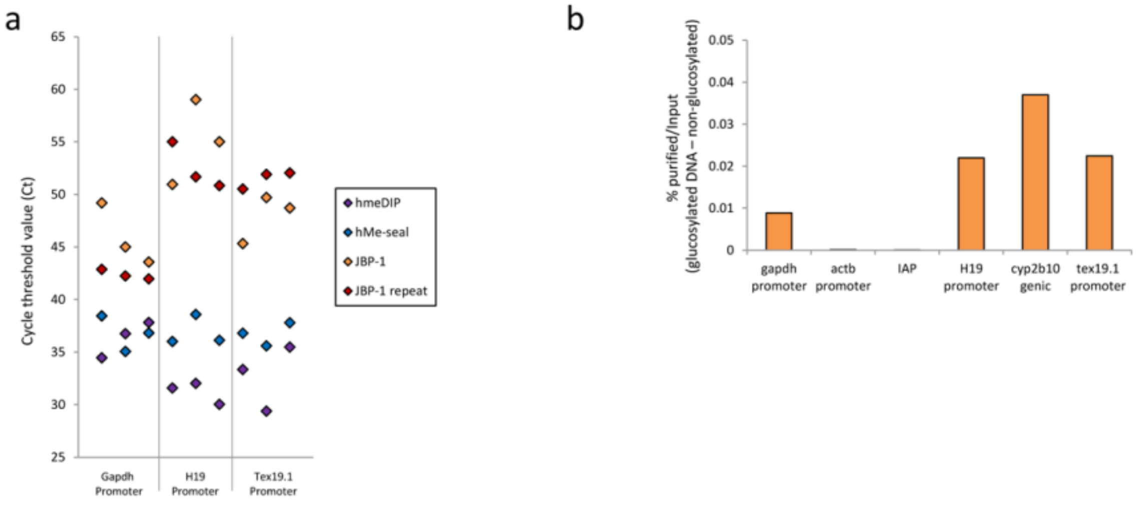

S2. JBP-1 affinity purification of 5hmC results in lower DNA yield than alternative techniques but does return similar patterns of 5hmC enrichment. **(a)** Cycle threshold plots of individual qPCR amplified samples over 3 loci (5hmC negative: *Gapdh*, 5hmC positive: *H19* and *Tex19.1* promoters) in the mouse liver. Triplicates are plotted to show technical replicates of amplification whilst for JBP-1 a separate enrichment was carried out to ensure the low levels of DNA returned was not due to a kit malfunction. JBP-1 purification 1 = yellow, JBP-1 purification 2 = red, hmeDIP = purple, hMe-seal = blue. **(b)** Non-normalised data following qPCR amplification of JBP-1 enriched DNA. Non-specific background was removed by subtracting the amplification values of DNA which had not been glucosylated from that which did. Plots are shown relative to the amplification of input DNA (% purified / input each following background subtraction). Although the patterns look encouraging the absolute levels of enrichment are extremely low (all <0.04% of the input).

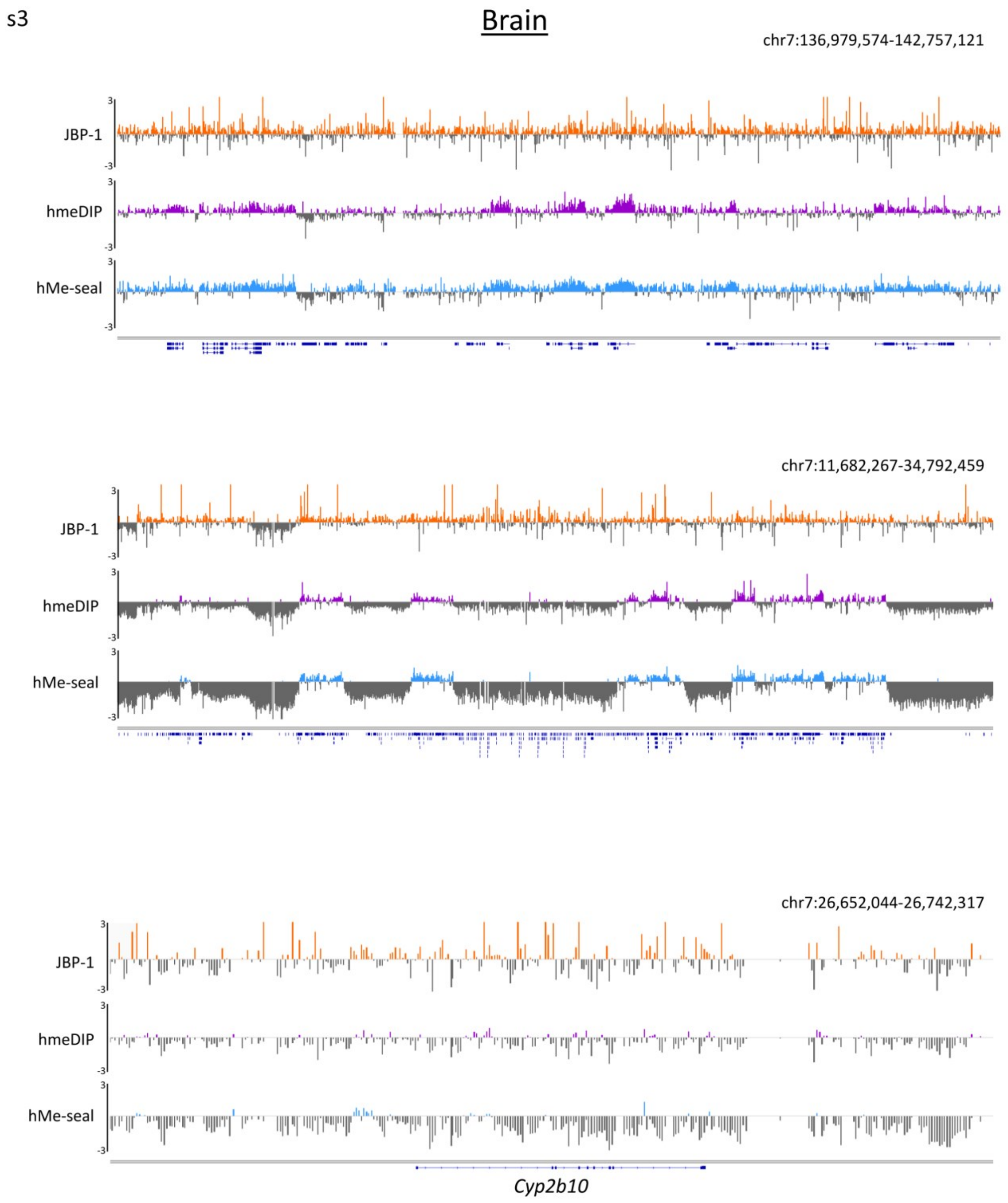

S3. Microarray patterns of 5hmC enrichment following JBP-1 purification reveal high levels of background noise. Data plotted on log2 scale from +3 to -3. Coordinates of the three loci are displayed on the right. JBP-1 enrichment = orange, hmeDIP enrichment = purple, hMe-seal enrichment = blue, depletion = grey, gene structures = blue bars.

s4

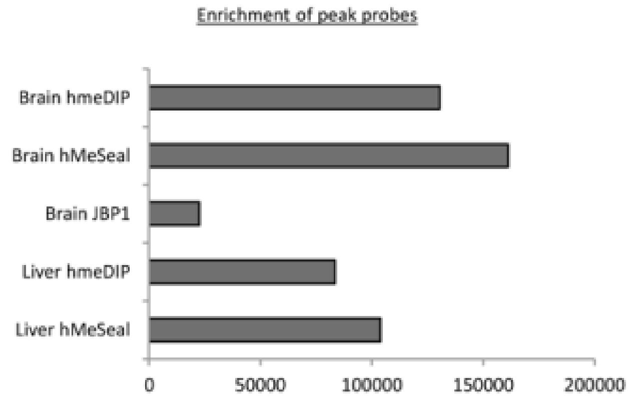

S4. Plot of total number of 5hmC enriched “peak probes” found following hmeDIP, hMe-seal or JBP-1 affinity purification in brain and liver tissues.

s5

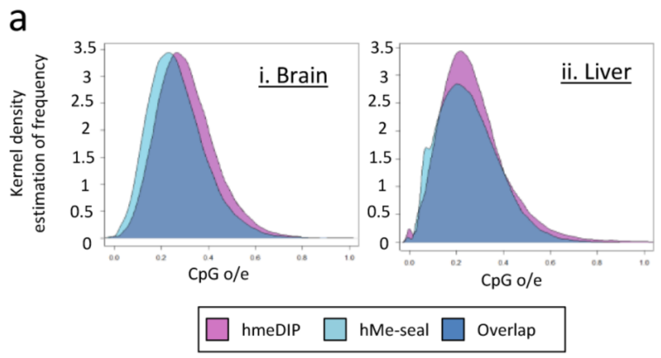

S5. Kernel distribution plots of CpG observed /expected (o/e) ratios at 5hmC enriched peak probes in the mouse brain (i) and the liver (ii). The number of peak probes containing a particular CpG o/e ratio is shown for both hmeDIP (purple) and hMeSeal (light blue) datasets as well as for the overlap (dark blue).

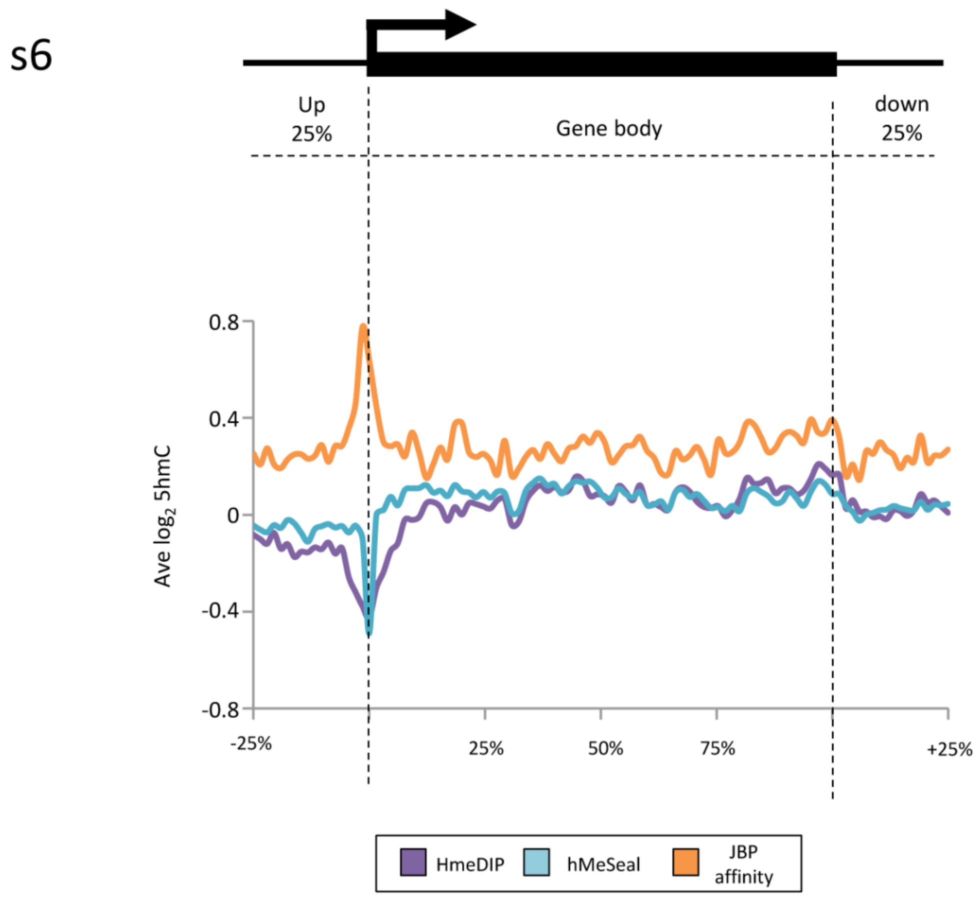

S6. Average pattern of 5hmC across mouse brain promoter and genic regions are highly similar following independent purification by either hmeDIP (purple) or hMeSeal (blue) but completely unique following JBP-1 purification (orange) peaking directly over the TSS. Average 5hmC values were calculated at positions relative to the full length of the gene with analysis extending 25% up and downstream. Y-axis plots average log2 scores taken from microarray datasets. Figure directly related to figure 2a.

s7

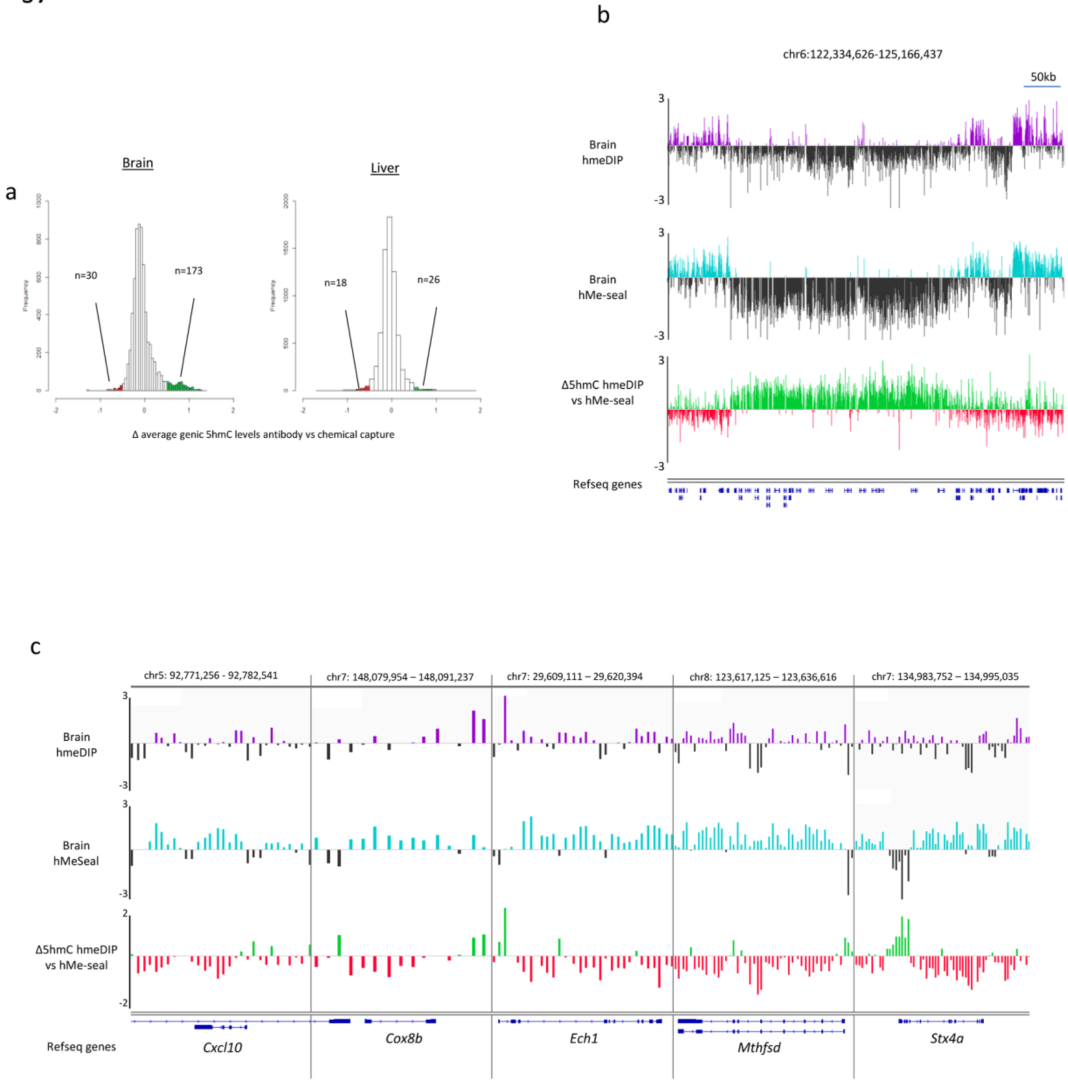

S7. Identification of genes showing technique dependant 5hmC enrichment bias. **(a)** Histograms showing the levels of 5hmC enrichment bias across all of the genes following length normalisation. The vast majority of the genes did not differ by more than log2 0.5 fold across the two techniques. Genes with greater than log2 0.5 fold changes in either direction were selected for further analysis. Red bars = genes showing hMe-seal enrichment (n=30), green bars = hmeDIP enrichment (n-173). **(b)** Genome browser visualisation reveals that many of the hmeDIP enriched genes are found over regions of low 5hmC, indicating higher levels of noise is introduced by the antibody based approach. All plots are on log2 scale between +3 and -3. Purple bars = hmeDIP enrichment, blue= hMe-seal enrichment, grey = 5hmC depletion, green= hmeDIP specific 5hmC bias, red= hMe-seal specific bias. Genes denoted below are blue bars. **(c)** Examples of hMe-seal specific enriched genes. Genome browser visualisation of brain microarray datasets. Coordinates are given above each region. All plots are on log2 scale between +3 and -3. Purple bars = hmeDIP enrichment, blue= hMe-seal enrichment, grey = 5hmC depletion, green= hmeDIP specific 5hmC bias, red= hMe-seal specific bias. Genes denoted below are blue bars.

s8

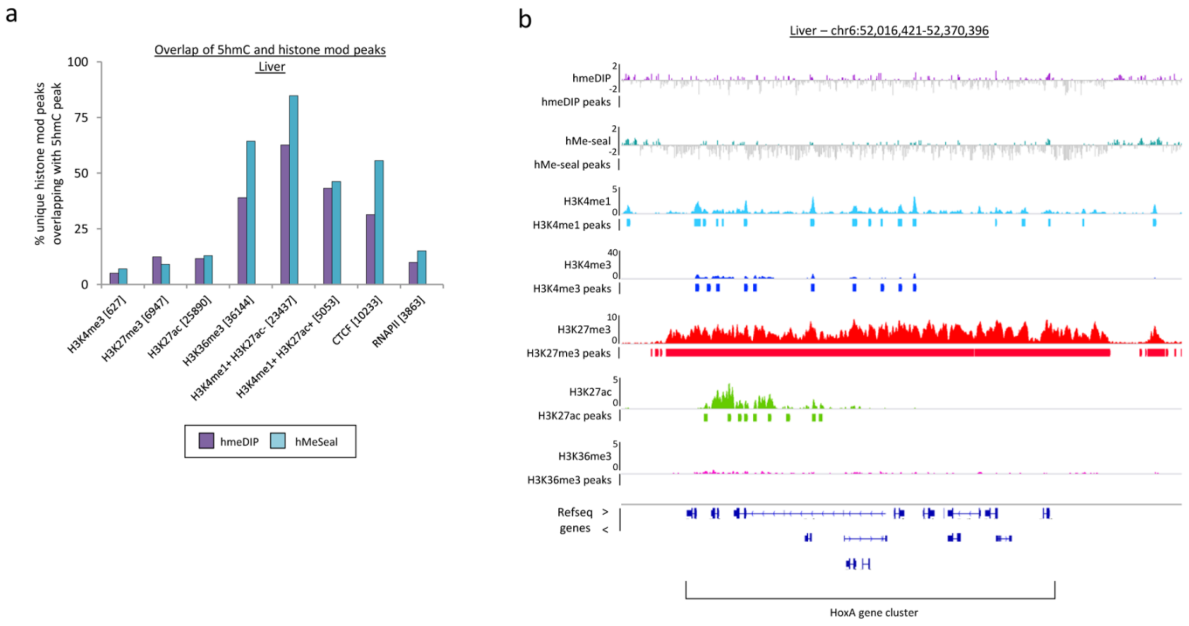

S8. Regions of 5hmC enrichment are associated with select histone modifications in the mouse liver. Figure relates to Figure 3.**(a)** Overlap between peaks of histone modifications/DNA binding proteins with peaks of 5hmC derived through the three purification techniques in the mouse liver. Plots show percentage of total histone modification peaks which overlap with a peak of 5hmC by at least 1 base pair. Total number of histone modification peaks are shown below in square brackets. **(b)** Genome browser visualisation of 5hmC patterns (hmeDIP: purple, hMeSeal: teal, JBP-1 affinity: orange) overlap with select histone modifications (H3K4me1:light blue , H3K4me3: dark blue, H3K27me3: red, H3K27ac: green, H3K36me3: pink) in the mouse liver. Array data plotted on log2 scale whilst ChIP-seq the number of reads. Refseq genes are displayed below.

s9

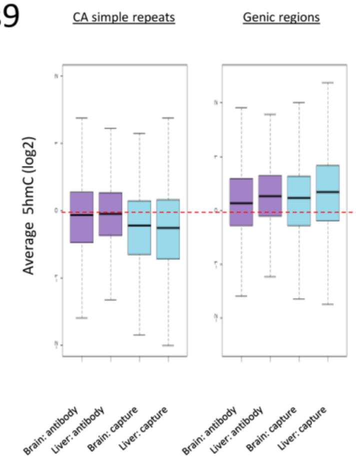

S9. Boxplot of 5hmC enrichments at regions of CA simple tandem repeats versus genic regions rich in 5hmC. Although showing bias to these simple tandem repeats the levels of 5hmC here even in hmeDIP are lower than at enriched loci such as gene bodies. Y-axis= average log2 5hmC level for all probes mapping to feature. Purple boxes = hmeDIP , blue boxes= hMe-seal.

s10

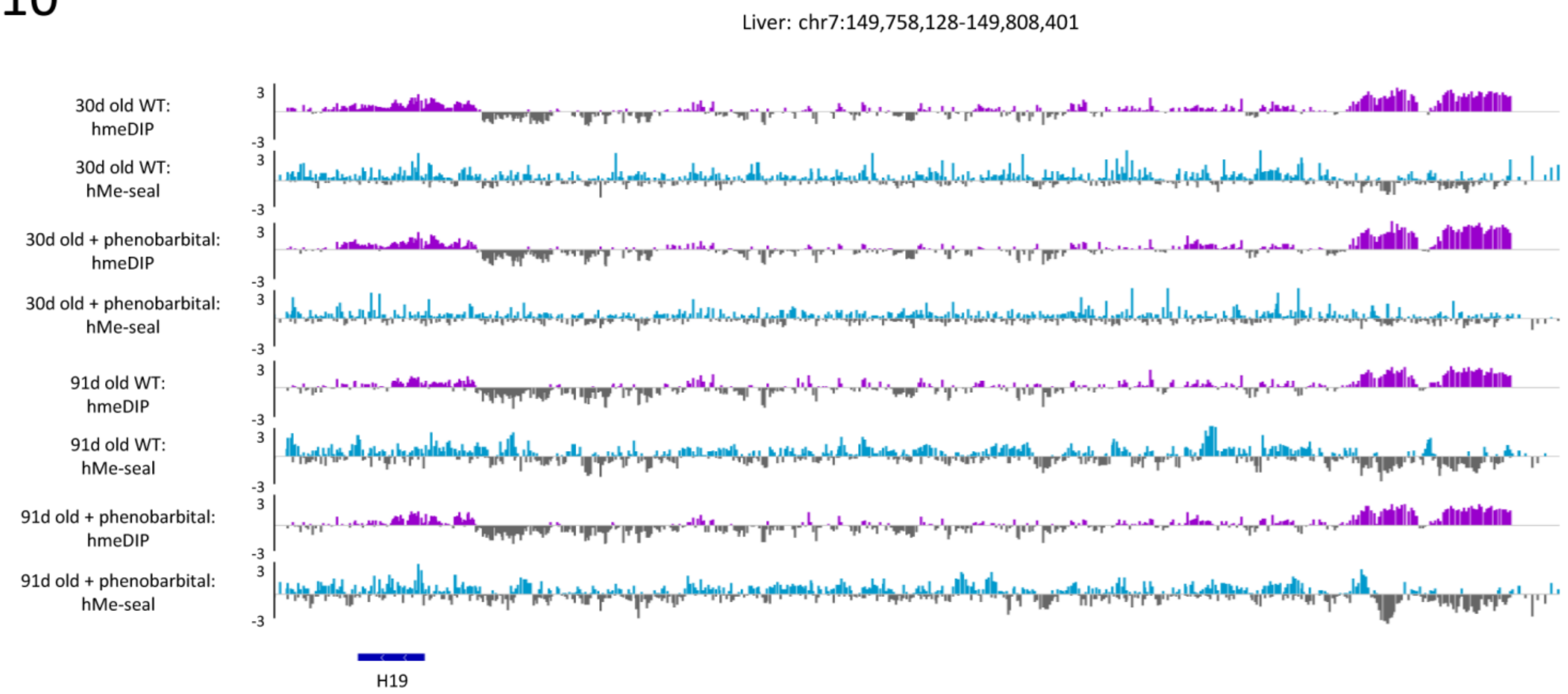

S10. Identified technique dependant 5hmC bias is seen in earlier reported studies. Data from published work investigating epigenetic changes in the liver which occur in response to the drug phenobarbital (25) in a different strain of mouse also show the same patterns of 5hmC bias over the *H19/Igf2r* locus. Both young (30day old) and old (91 day old) WT and drug treated mice show the same levels of antibody specific bias. Purple bars = hmeDIP enrichment, blue= hMe-seal enrichment, grey = 5hmC depletion, structure of the *H19* gene is shown in blue below.

s11

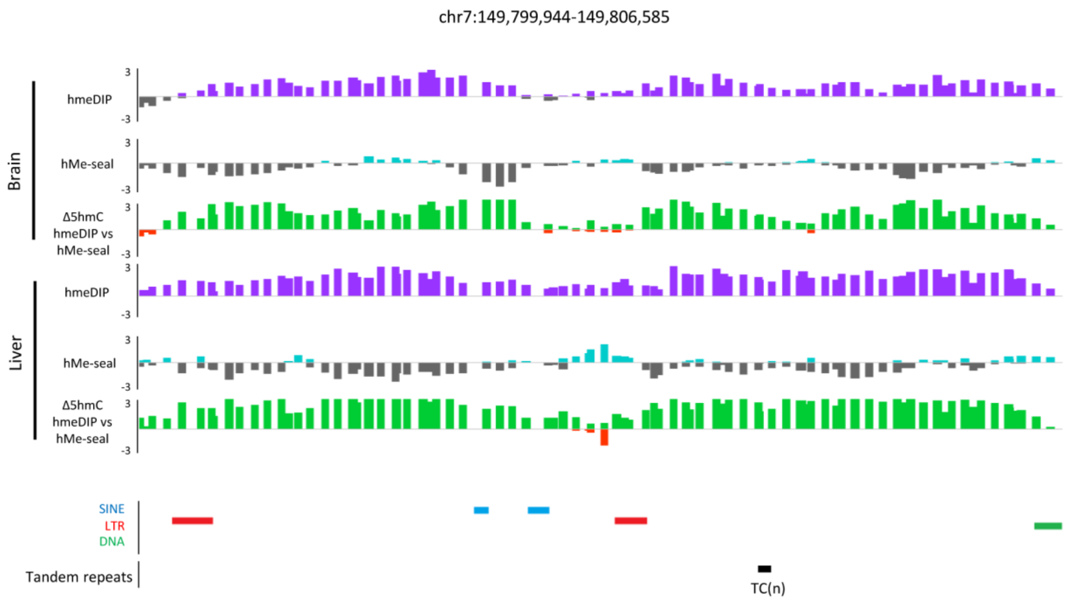

S11. The strong region of technique dependant enrichment between *H19* and *Igf2r* corresponds to several repetitive elements as well as a region of TC tandem repeats. Purple bars = hmeDIP enrichment, blue= hMe-seal enrichment, grey = 5hmC depletion, green= hmeDIP specific 5hmC bias, red= hMe-seal specific bias, SINEs= blue boxes, LTRs = red boxes, DNA repeat elements = green box, TC simple tandem repeats = black box.

s12

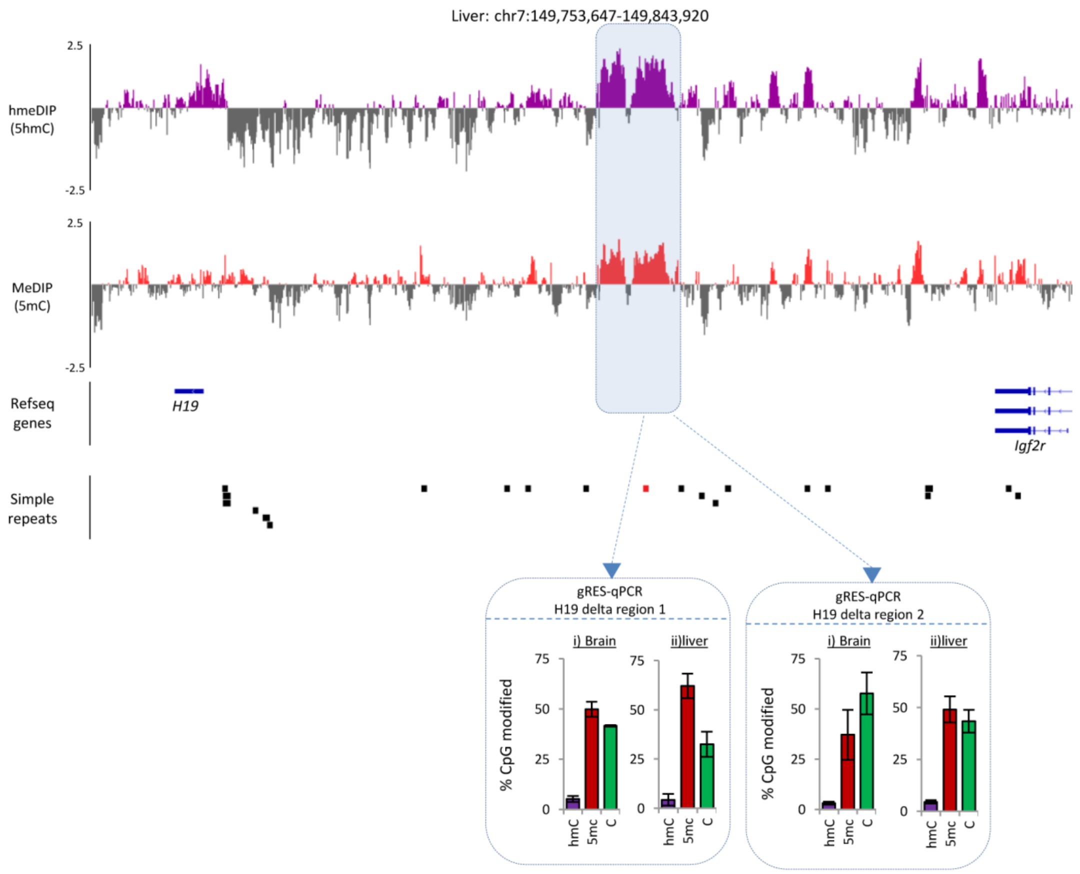

S12. Profiles of 5mC enriched liver DNA over the *H19/Igf2r* locus reveals similarly high levels of the modification between the two genes. Unlike 5hmC, this elevation in 5hmC can be independently validated by gRES-qPCR (boxed region below). Purple tracks = hmeDIP enrichment, red= MeDIP enrichment, grey = 5hmC or 5mC depletion. Genes are displayed as blue bars below with simple repeats shown as black bars. Boxed highlighted area denotes regions tested by gRES-qPCR. Boxed bar plots represent the percentage of each modification at a single CpG in the sequence CCGG following normalisation (purple; 5hmC, red; 5mC, green; C). Error bars display the standard error of the biological replicates.

Primers

qPCR primers	Fw	Rev
Gapdh Promoter	CCACTCCCCTTCCCAGTTTC	CCTATAAATACGGACTGCAGC
Act B promoter	ATGTACAGGAATAGCCTCCG	CTTAAGTGCTCGATATCCAC
IAP	CGTGAGAACGCGTCGAATAA	TTCTGGTTCTGGAATGAGGG
H129 Promoter	GCCAAGAGAGAAGAAGGAGA	GAATGTTGAAGGACTGAGGG
Cyp2b10 genic	TAAGTCCCATCCCTCTGTTC	GACAGACCCCATCTCAAAAA
Tex191.1 promoter	GGGAGATATGTAAATGAGCTGG	CATCCTTACCTCCCTGACTGAG

gRES-qPCR primers	Fw	Rev
gRES-gapdh Promoter	CCACTCCCCTTCCCAGTTTC	CCTATAAATACGGACTGCAGC
gRES-Muc6	GAGATGCCTGTTGTCCTAGA	CTACCTTTCTCACCCTTCCT
gRES-Mill1	TCTAGGGAGGGTAGATTGGA	CTGGGCAGGATAGTTTCAGA
gRES-Nanos2	CTCTGCTCCACAAAACTCCT	TACCTCACCCTCCTGTCTTT
gRES-Tfdp1	ACAGCAGCCACAACACTTGG	CAAGGTCTTACTCTAGGATTGA
gRES-H19 promoter	GCCAAGAGAGAAGAAGGAGA	GAATGTTGAAGGACTGAGGG
gRES-H19 delta 1	CAAACACCCAGCAGAAACCA	CTCCAACAAAACTCTCGTCC
gRES-H19 delta 2	TTGTGCATAGTGTCTTCATG	TGAGGCAGAGTGAGAAGAGA
gRES-Tsix/Xist delta 1	CTTCTGTCTTCTACTTGGGC	TGTGTGTCATGTGTGAGAGG
gRES-Tsix/Xist delta 2	CACACTAATACGAGCACTCC	CACAGCGGACTGGATAAAAG
